# Characterization of High-Risk-Other Human Papillomavirus Genotypes in Papanicolaou Tests, High-Grade Squamous Intraepithelial Lesions, and Cervical Cancer

**DOI:** 10.31486/toj.24.0018

**Published:** 2024

**Authors:** Caitlin E. Witt, Elizabeth F. Sutton, Ashley M. Stansbury, Ashley N. Winters, Luke C. Konur, Meng Luo, Jennifer E. Cameron, Beverly Ogden

**Affiliations:** ^1^ Department of Obstetrics and Gynecology, Louisiana State University Health Sciences Center, New Orleans, LA; ^2^ Department of Research, Woman's Hospital, Baton Rouge, LA; ^3^ Department of Pathology, Woman's Hospital, Baton Rouge, LA; ^4^ Department of Microbiology, Immunology and Parasitology, Louisiana State University Health Sciences Center, New Orleans, LA

**Keywords:** *Genotype*, *human papillomavirus viruses*, *neoplasms*, *uterine cervical neoplasms*, *vaccination*

## Abstract

**Background:** The objective of this study was to determine the human papillomavirus (HPV) genotypes of high-risk-other HPV Papanicolaou (Pap) tests and of biopsy tissues from patients with high-grade squamous intraepithelial lesion (HGSIL) or cervical cancer. High-risk-other HPV status was determined with the cobas HPV Test (Roche Diagnostics, North America) that identifies 12 high-risk, non-16/18 HPV genotypes. We hypothesized that we would find genotypes of HPV in our population that are not covered by the 9-valent HPV vaccine.

**Methods:** For this retrospective cohort study, we randomly selected 50 high-risk-other HPV Pap test samples from 2018 from our pathology department registries for HPV genotype determination by Roche Linear Array (Roche Diagnostics, North America). Then we randomly selected 76 cervical biopsy samples of HGSIL or cervical cancer with high-risk-other HPV or HPV unknown status from 2016 to 2022 for HPV genotype determination by next-generation sequencing. Results are reported as counts and frequencies.

**Results:** In the 50 high-risk-other HPV Pap test samples, 21 genotypes of HPV were noted; the most common were 53 (n=6), 51 (n=6), and 59 (n=5). In the samples with HGSIL or cervical cancer, 16 HPV genotypes were detected; the most common were 16 (n=26), 58 (n=12), and 33 (n=8). Among the patients with HGSIL or cervical cancer, the 9-valent HPV vaccine provided coverage for all the HPV variants found in 88% of patients, partial coverage in 8% of patients, and no coverage in 4% of patients.

**Conclusion:** The 3 most common HPV genotypes seen in our high-risk-other HPV Pap test samples are not covered by the 9-valent HPV vaccine. For the HGSIL and cancer samples, 88% of the samples had full HPV genotype coverage with the 9-valent HPV vaccine. This study highlights a presence of HPV that will not be protected by vaccination in a high-risk population.

## INTRODUCTION

According to the Centers for Disease Control and Prevention and the World Health Organization, prevention of cervical cancer through vaccination against the human papillomavirus (HPV) prior to HPV exposure is the gold standard in cervical cancer prevention.^1^ The 9-valent HPV vaccine (GARDASIL 9, Merck & Co., Inc) targets HPV genotypes 6, 11, 16, 18, 31, 33, 45, 52, and 58, the genotypes estimated to account for 90% of cervical cancer cases globally.^2^

HPV genotype prevalence has been shown to vary with ethnicity and race. In a study by Montealegre et al, non-Hispanic Black women (36%) and Hispanic women (42%) were more likely to test positive for an HPV genotype not protected by any HPV vaccine (bivalent, quadrivalent, and nonavalent) compared to non-Hispanic White (24%) and Asian (16%) women.^3^

HPV prevalence also varies based on geographic location, but geographic understanding of specific HPV genotypes is limited and remains a substantial gap in knowledge.^2^ Geographic differences in cervical cancer diagnosis persist in the United States, with the South having the highest incidence of new cervical cancer diagnoses.^4,5^ The need to understand the HPV genotypes that precede cervical cancer cases is critical, particularly because of these regional differences.

Knowledge gaps about the prevalence and distribution of HPV genotypes are in part attributable to nonspecific clinical test results; positive samples collected via Papanicolaou (Pap) testing are reported as only HPV 16, HPV 18, and HPV other. We determine high-risk-other HPV status with the cobas HPV Test (Roche Diagnostics, North America) that identifies 12 high-risk non-16/18 HPV genotypes. We hypothesized that we would find HPV genotypes in our population that are not covered by the 9-valent HPV vaccine. To test our hypothesis, we selected 50 HPV high-risk-other HPV Pap test samples and 76 biopsy samples from patients with high-grade squamous intraepithelial lesion (HGSIL) or cervical cancer for HPV genotype determination.

## METHODS

This study was determined to be exempt by the Woman's Hospital Foundation Institutional Review Board (RP-22-004, March 21, 2022).

### Sample Selections

This retrospective study was conducted in Louisiana using Pap test and cervical biopsy samples from registries in the Pathology Department at Woman's Hospital in Baton Rouge, Louisiana. Our institution uses the cobas HPV Test to co-test Pap tests for HPV genotypes. The cobas HPV Test provides results for HPV 16, HPV 18, and high-risk-other HPV, a category that includes genotypes 31, 33, 35, 39, 45, 51, 52, 56, 58, 59, 66, and 68.

During 2018, 5,774 HPV tests were performed at the study site, and 22% were HPV positive. Of those positive results, 87% were high-risk-other HPV. We conducted a pilot study with 50 randomly selected high-risk-other HPV Pap test samples (with stored samples available for additional testing) for HPV genotype determination as described in the HPV Genotyping section. Population demographics were not available for this cohort.

**Figure 1.**
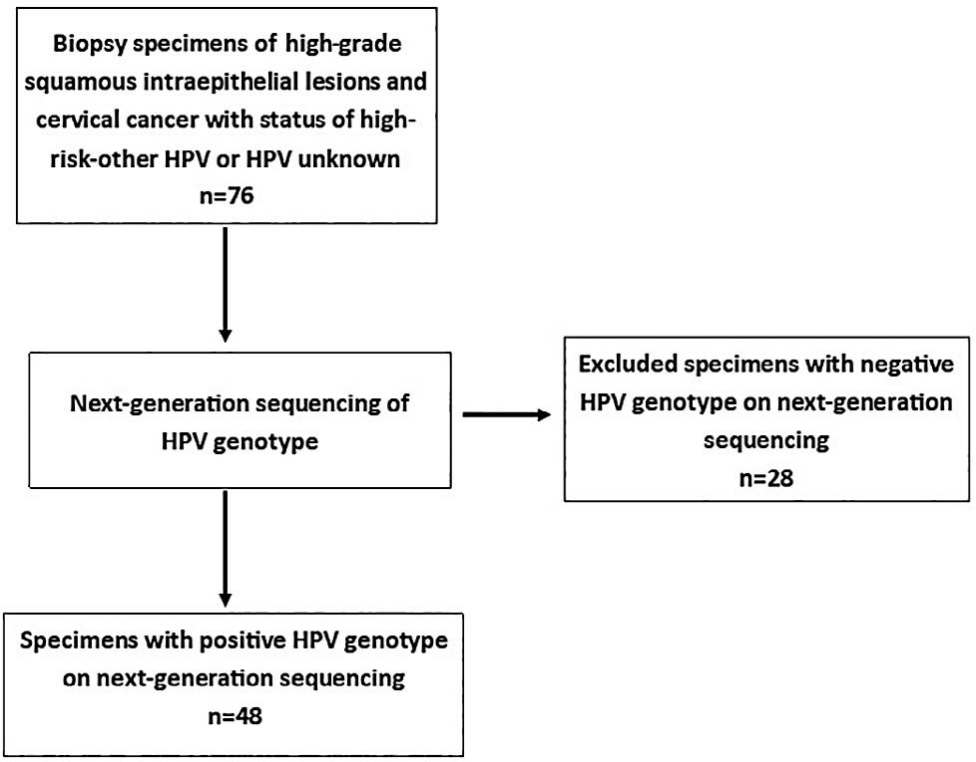
Workflow of cervical biopsy tissue specimens selected for human papillomavirus (HPV) genotype testing by next-generation sequencing.

The pilot study of Pap test samples informed our study to investigate HPV genotypes in archived cervical samples from patients with HGSIL or cervical cancer from the same institution. From the cervical tissue registry, 76 cervical biopsy tissues with either high-risk-other HPV status according to the cobas HPV Test or HPV unknown status were randomly selected for next-generation sequencing for HPV genotypes. Samples were formalin-fixed, paraffin-embedded (FFPE) cervical biopsy tissues with histopathology consistent with HGSIL or cervical carcinoma collected between 2016 and 2022. Sample selection was also based on pathology diagnosis and the availability of archived tissue of adequate size. Of the 76 samples selected, 48 are included in this report. Twenty-eight cases were excluded because next-generation sequencing showed no HPV results (Figure 1).

### HPV Genotyping

For the Pap test samples, whole genomic DNA was isolated from liquid cytology using the QIAamp DNA Blood Kit (QIAGEN). FFPE tissues were sectioned in 10 μm ribbons and treated with deparaffinization solution (QIAGEN, Zymo Research Corporation). Whole genomic DNA was isolated using the QIAamp DNA FFPE Tissue Kit (QIAGEN) or the Quick-DNA FFPE Miniprep Kit (Zymo Research Corporation). Purity and concentration of DNA extracts were determined by ultraviolet spectrophotometry, and extracts were stored at –20 °C. Specimen DNA extracts were used as a template in multiplex polymerase chain reaction (PCR) that included primers for human beta-globin and PGMY09/11 primers for HPV. Amplicons were denatured and hybridized to linear array strips using the Roche Linear Array (Roche Diagnostics, North America) that contains probes for 37 HPV genotypes and human beta-globin. Probe bands were scored positive according to the manufacturer's protocol. The 37 HPV genotypes are low-risk (6, 11, 26, 40, 42, 53, 54, 55, 61, 62, 64, 66, 67, 69, 70, 71, 72, 73, 81, 82, 83, 84, IS39 [variant of 82], and CP6108 [89]) and high-risk (16, 18, 31, 33, 35, 39, 45, 51, 52, 56, 58, 59, and 68).

For the tissue samples, HPV genotyping was performed by next-generation sequencing. HPV libraries were prepared by PCR amplification of genomic DNA using MY09/11 HPV L1 primers. Libraries were barcoded and sequenced using a MiSeq instrument and reagent kits (Illumina, Inc). The data analysis pipeline was similar to HPV-QUEST described by Yin et al.^6^ Briefly, raw sequencing reads were filtered to remove poor-quality reads and indices were decoded. After trimming to remove barcoding and adapter sequences, reads were aligned to a catalog of HPV genotype reference sequences. Data output included HPV genotypes and aligned read counts for each specimen.

The following definitions were applied: (1) adequate sample, detection of human beta-globin; (2) high-risk HPV, based on the IARC (International Agency for Research on Cancer)-12, HPV 16, 18, 31, 33, 35, 39, 45, 51, 52, 56, 58, and 59; (3) probable carcinogenic genotypes, HPV 68, 73, and 82; and (4) HPV genotypes in the GARDASIL 9 vaccine, HPV 6, 11, 16, 18, 31, 33, 45, 52, 58.^7^ All other HPV genotypes detected were scored as low/unknown risk. All specimens passed quality control and were included in the report.

## RESULTS

### Pilot Study: HPV Genotypes in Pap Test Samples

**Figure 2.**
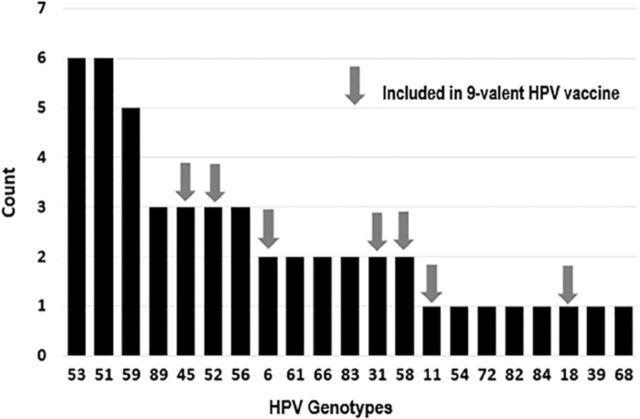
Human papillomavirus (HPV) genotypes in Papanicolaou test samples detected by Roche Linear Array (Roche Diagnostics, North America) hybridization assay.

Of the 50 high-risk-other HPV Pap test samples analyzed, 16 (32%) had 1 high-risk HPV genotype detected, 12 (24%) had multiple high-risk HPV genotypes detected, 1 (2%) had a low-risk HPV genotype detected, and 21 (42%) had no HPV genotype detected. In the test samples in which HPV was detected, 21 different genotypes of HPV were noted, with the most common being 53 (n=6), 51 (n=6), and 59 (n=5) (Figure 2). Fourteen genotypes were identified that are not covered by the 9-valent HPV vaccine (Figure 2).

### High-Grade Squamous Intraepithelial Lesion and Cervical Cancer Population Characteristics

Of the 48 tissue samples, 58% were from patients of the White race, 35% from the Black race, and 6% from other races (Table 1). Ethnically, 4% were Hispanic, and 96% were non-Hispanic. The mean age of the patients was 38.7 years, and their average body mass index at the time of diagnosis was 32.1 kg/m^2^ (Table 2). Thirty-eight percent of patients were current tobacco smokers, and 6% had a history of smoking. Thirty-three percent of the population had private insurance, 65% were Medicaid-eligible or self-pay, and 2% were Medicare-eligible. Of the population, 65% had a prior high-risk-other HPV result in their medical record, and 35% had HPV unknown status. The cervical pathology of this population included 52% with HGSIL, 40% with squamous cell carcinoma, and 8% with adenocarcinoma.

**Table 1.** Demographics and 9-Valent Human Papillomavirus Vaccine Coverage by Race, Ethnicity, and Overall for Patients With High-Grade Squamous Intraepithelial Lesion or Cervical Cancer, n=48

Variable	Overall	Full Vaccine Coverage	Partial Vaccine Coverage	No Vaccine Coverage
Race				
White	28 (58)	25 (89)	3 (11)	0
Black	17 (35)	14 (82)	1 (6)	2 (12)
Other	3 (6)	3 (100)	0	0
Ethnicity				
Hispanic	2 (4)	2 (100)	0	0
Non-Hispanic	46 (96)	40 (87)	4 (9)	2 (4)
Overall	48 (100)	42 (88)	4 (8)	2 (4)

Notes: Data are presented as n (%). Percentages for vaccine coverage are calculated across rows.

**Table 2.** Population Demographics for Patients With High-Grade Squamous Intraepithelial Lesion or Cervical Cancer, n=48

Variable	Value
Age, years, mean ± SD	38.7 ± 11.0
Weight, kg, mean ± SD	84.7 ± 24.2
Body mass index, kg/m^2^, mean ± SD	32.1 ± 9.0
Smoking status	
Current	18 (38)
Former	3 (6)
Never	24 (50)
Missing	3 (6)
Insurance	
Private	16 (33)
Medicaid/self-pay	31 (65)
Medicare	1 (2)
Human immunodeficiency virus status	
Positive	2 (4)
Negative	32 (67)
Missing	14 (29)
Human papillomavirus status	
High-risk-other	31 (65)
Unknown	17 (35)
Diagnosis	
High-grade squamous intraepithelial lesion	25 (52)
Squamous cell carcinoma	19 (40)
Adenocarcinoma	4 (8)

Note: Data are presented as n (%) unless otherwise indicated.

### HPV Genotypes in High-Grade Squamous Intraepithelial Lesion and Cervical Cancer Samples

**Figure 3.**
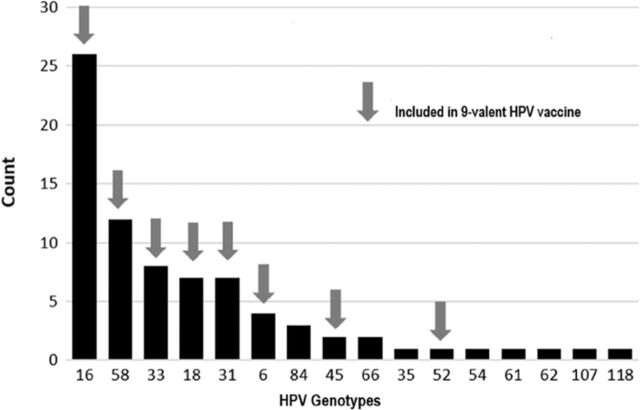
Human papillomavirus (HPV) genotypes in high-grade squamous intraepithelial lesion and cervical cancer biopsy tissues detected by next-generation sequencing.

**Figure 4.**
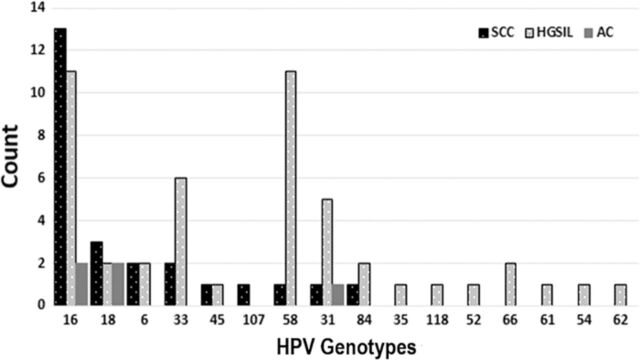
**Human papillomavirus (HPV) genotypes stratified by histopathology diagnosis.** AC, adenocarcinoma; HGSIL, high-grade squamous intraepithelial lesion; SCC, squamous cell carcinoma.

Of the 48 cervical samples with HGSIL or cervical cancer analyzed, 16 different HPV genotypes were detected (Figure 3). The most common HPV genotype result was 16 (n=26), followed by 58 (n=12) and 33 (n=8) (Figure 3). Eight HPV genotypes were found that are not included in the HPV vaccine: 84, 66, 35, 54, 61, 62, 107, and 118. Forty-two percent of the samples had more than 1 HPV genotype. Eleven individuals had 2 HPV genotypes, 8 individuals had 3 HPV genotypes, and 1 individual had 6 HPV genotypes. The individual with 6 HPV genotypes was human immunodeficiency virus–positive with HPV genotypes 16, 18, 31, 45, 54, and 62. HPV genotypes 16, 18, and 31 were seen in the 4 cases of adenocarcinoma (Figure 4). The most common genotype of squamous cell carcinoma was 16 (n=13) followed by 18 (n=3).

Notably, among HGSIL/cancer cases, 88% had full coverage by the 9-valent HPV vaccine, 8% had partial coverage, and 4% had no coverage of their HPV genotypes (Table 1). Also of note, HPV genotypes 107 and 118 were detected in 1 specimen each by next-generation sequencing. These 2 genotypes are not included in available HPV clinical tests and would likely have been missed by most probe-based assays, including the Roche Linear Array.

## DISCUSSION

Our study investigated local HPV genotypes in 2 settings. The 3 most common HPV genotypes detected in the high-risk-other Pap test samples (51, 53, and 59) are not covered by the current 9-valent HPV vaccine. However, the 3 most common HPV genotype results from the cervical HGSIL and cancer samples (16, 58, and 33) are covered by the 9-valent HPV vaccine. HPV 84, 66, 35, 54, 61, 62, 107, and 118 were seen in the HGSIL/cancer group and are not covered by the HPV vaccine. These results show that the most common HGSIL/cancer HPV genotypes found in this study are within coverage of an HPV vaccine. However, HPV genotypes are present that are not covered with HPV vaccination. Forty-two percent of individuals with HGSIL or cancer had a co-occurrence of multiple HPV infections.

While the vaccination status of our sample was unknown, in 2022 in Louisiana, 67.3% of teens aged 13 to 17 years were reported as being up to date with their HPV vaccination.^8^ Across the United States in 2022, 62.6% of teens aged 13 to 17 years were up to date on HPV vaccination status.^8^

The United States has large geographic differences in cervical cancer rates, and these differences have persisted even as cervical cancer incidence and mortality have declined with cervical cancer screening.^4^ Data describing the distributions within the United States of HPV genotypes in cervical cancer are limited. One study that examined HPV in precancer and cancer diagnosed in New Mexico found the greatest risk of invasive cervical cancer with HPV 16, 18, 33, 35 and 45.^9^ Our study reveals infections with HPV genotypes in HGSIL and cervical cancer that will not be protected by vaccination in a high-risk southern United States population. Emphasis on increasing access to vaccination and screening will have the highest impact on improving prevention of cervical cancer; however, the benefit of a more inclusive HPV vaccine is not currently understood.^10^

Mix et al conducted a study of HPV genotypes attributing to precancerous and cervical cancer lesions and found a higher proportion of precancerous lesions caused by HPV not covered in the 9-valent HPV vaccine among non-Hispanic Black women compared to non-Hispanic Asian/Pacific Islander, non-Hispanic White, and Hispanic women.^11^ Consequently, the Mix et al study anticipated a lower rate of prevention of precancerous cervical lesions with the 9-valent HPV vaccine for non-Hispanic Black women.^11^ In our study, the 2 (4%) individuals with no coverage of HPV genotype by the 9-valent HPV vaccine were Black. Of the 4 (8%) individuals with partial vaccine coverage, 1 individual was Black and 3 were White. The 2 individuals with Hispanic ethnicity in this study had 100% coverage by the 9-valent HPV vaccine.

While the most oncogenic HPV genotypes are well established, further investigation of the impact of high-risk HPV genotypes on cervical health is warranted. Clinically, these data are important because HPV genotype is used to guide screening recommendations and follow-up of abnormal Pap test results. With increased understanding of the HPV distribution among different races and geographic regions, an updated HPV triage algorithm could improve the equity of cervical cancer screening.^10^

Our study has several strengths and limitations. Strengths of this study include a racially diverse population with a diverse insurance status. The initial HPV test used for selection into the study is an accredited HPV test. The Roche Linear Array is a widely published method for HPV genotype surveillance, and next-generation sequencing enables comprehensive genotype discovery. A limitation of the study is the small sample size, which was due to inadequate tissue volume available for testing for many archived specimens. The study was underpowered to perform statistical analysis across demographic strata.

## CONCLUSION

This study demonstrates the importance of continued investigation of HPV genotypes, especially in high-risk populations in at-risk geographic areas. Further research is needed to determine if different screening and follow-up recommendations are needed in high-risk populations or populations with higher rates of unprotected HPV genotypes. Surveillance of HPV genotype prevalence among women in different geographic regions and with different racial and ethnic backgrounds will be important to ensure adequate cervical cancer prevention strategies are deployed, including appropriate screening tests and vaccine formulas.

## Notes

*This article meets the Accreditation Council for Graduate Medical Education and the American Board of Medical Specialties Maintenance of Certification competencies for Patient Care, Medical Knowledge, and Practice-Based Learning and Improvement.*
